# Sex-Specific Patterns and Predictors of Reverse Left Ventricular Remodeling and Outcomes in STEMI Patients with LVEF ≤ 50% After Successful Primary Angioplasty

**DOI:** 10.3390/biomedicines13071782

**Published:** 2025-07-21

**Authors:** Bogdan-Flaviu Buz, Sergiu-Florin Arnautu, Mirela-Cleopatra Tomescu, Minodora Andor, Simina Crisan, Dan Gaita, Cristina Vacarescu, Constantin-Tudor Luca, Cristian Mornos, Dragos Cozma, Diana-Aurora Arnăutu

**Affiliations:** 1 Doctoral School, Faculty of Medicine, Victor Babes University of Medicine and Pharmacy, 300041 Timisoara, Romania; flaviu-bogdan.buz@umft.ro (B.-F.B.); andor.minodora@umft.ro (M.A.); 2 Multidisciplinary Heart Research Center, Victor Babes University of Medicine and Pharmacy, 300041 Timisoara, Romania; aurora.bordejevic@umft.ro; 3 Department of Internal Medicine, Victor Babes University of Medicine and Pharmacy, 300041 Timisoara, Romania; 4 Timisoara Municipal Clinical Emergency Hospital, 300040 Timisoara, Romania; 5 Department of Cardiology, Victor Babes University of Medicine and Pharmacy, 300041 Timisoara, Romania; simina.crisan@umft.ro (S.C.); cristina.vacarescu@umft.ro (C.V.); constantin.luca@umft.ro (C.-T.L.); dragos.cozma@umft.ro (D.C.); 6 Timisoara Institute of Cardiovascular Diseases, 13 Gh. Adam Street, 300310 Timisoara, Romania; dan.gaita@umft.ro (D.G.); mornos.cristian@umft.ro (C.M.)

**Keywords:** STEMI, left ventricular remodeling, myocardial work, sex differences, global work efficiency, echocardiography, major adverse cardiac events, Cox regression

## Abstract

**Background**: Sex-related differences in left ventricular (LV) reverse remodeling following ST-segment elevation myocardial infarction (STEMI) remain underexplored. We aimed to investigate predictors of reverse remodeling and its association with clinical outcomes, with a focus on sex-specific differences. **Methods**: We enrolled 253 STEMI patients (91 women, 28%) and assessed echocardiographic parameters at baseline and six months. LV reverse remodeling was defined as a ≥15% reduction in LV end-diastolic volume (LVEDV). Multivariate logistic regression identified independent predictors of remodeling. Clinical outcomes were evaluated over a median follow-up of 17 months (IQR 14–22 months), including major adverse cardiac events (MACEs). Kaplan–Meier and Cox regression analyses were performed. **Results**: Reverse remodeling occurred in 43% of patients and was more frequent in men than women (47% vs. 37%, *p* = 0.04). Male sex (OR 0.30; 95% CI: 0.14–0.65; *p* < 0.0001) and baseline global work efficiency (GWE) (OR 1.64; 95% CI: 1.45–1.85; *p* < 0.0001) were independent predictors. Men exhibited greater reductions in LVEDV, greater improvements in LV ejection fraction, and superior myocardial work indices. Over the follow-up, patients with reverse remodeling had significantly lower MACE rates compared to those without (10% vs. 24%, *p* < 0.01). Cox regression demonstrated that reverse remodeling was associated with a reduced risk of MACEs (HR 0.318; 95% CI: 0.181–0.557; *p* < 0.0001). **Conclusions**: LV reverse remodeling after STEMI is associated with improved clinical outcomes and is influenced by sex-specific differences. Baseline myocardial work indices, particularly GWE, are strong predictors of reverse remodeling. Men demonstrated a more favorable remodeling profile and myocardial recovery compared to women.

## 1. Introduction

ST-segment elevation myocardial infarction (STEMI) remains a leading cause of mortality and morbidity globally, despite considerable improvements in early reperfusion therapies such as primary percutaneous coronary intervention (PCI) [1,2]. The clinical course following STEMI is significantly influenced by the process of left ventricular (LV) remodeling, characterized by complex structural and functional changes in the myocardium. Adverse LV remodeling, encompassing ventricular dilatation, wall thinning, and a decline in ejection fraction, is a major predictor of subsequent heart failure and all-cause mortality [3,4]. Conversely, reverse remodeling, defined by a reduction in LV volumes and an improvement in left ventricular ejection fraction (LVEF), has emerged as a marker of myocardial recovery and improved clinical outcomes [5].

Patients presenting with reduced LVEF (≤50%) after or at the time of STEMI are at particularly high risk for adverse outcomes. Nonetheless, a subset of these patients undergoes significant reverse remodeling, which has been associated with reduced rates of heart failure hospitalization and better long-term survival [6]. However, predicting which patients will experience reverse remodeling remains a clinical challenge, owing to the heterogeneous nature of the remodeling process and its multifactorial determinants.

Recent research has highlighted that sex differences extend beyond the epidemiology and presentation of ischemic heart disease to influence myocardial healing and remodeling patterns [7,8]. Biological sex impacts the response to myocardial injury through mechanisms involving sex hormones, genetic expression, microvascular function, and inflammatory pathways [9,10]. Women have been shown to exhibit different patterns of myocardial remodeling compared to men, with some studies suggesting a more favorable remodeling profile, potentially attributed to estrogen-mediated cardioprotection and attenuated inflammatory responses [11,12]. However, the data are not uniform, and disparities in clinical outcomes between sexes persist, indicating that further investigation is warranted.

Moreover, sex-specific factors may also influence therapeutic responses post-STEMI, including the efficacy of pharmacological agents such as beta-blockers, angiotensin-converting enzyme inhibitors, and mineralocorticoid receptor antagonists, which are known to modulate the remodeling process [13]. Despite growing awareness of the impact of sex on cardiovascular disease processes, women have historically been underrepresented in clinical trials, leading to gaps in knowledge regarding sex-specific predictors of reverse remodeling and outcomes in the contemporary PCI era [14].

In this context, advanced imaging modalities such as speckle-tracking echocardiography (STE) have emerged as powerful tools for the assessment of myocardial function beyond conventional LVEF measurement. STE provides a quantitative evaluation of myocardial deformation through strain analysis, particularly global longitudinal strain (GLS), which has been shown to be a more sensitive marker of subclinical LV dysfunction and a stronger predictor of adverse remodeling and outcomes post-STEMI [15,16]. GLS quantifies the percentage of myocardial fiber shortening along the longitudinal axis, and reduced GLS has been associated with impaired myocardial mechanics and worse prognosis, even when LVEF is preserved.

More recently, myocardial work indices derived from non-invasive pressure–strain loops—integrating afterload into the assessment of myocardial deformation—have been introduced. Myocardial work parameters, including global work index (GWI), global constructive work (GCW), and global wasted work (GWW), offer a comprehensive evaluation of myocardial performance by accounting for the interaction between myocardial deformation and left ventricular pressure throughout the cardiac cycle [17,18]. These indices have shown promise in better reflecting myocardial energetics and efficiency, and may surpass traditional strain measurements in predicting functional recovery and adverse remodeling after STEMI. Large multicenter series published in the last five years confirm that global work efficiency (GWE) and global constructive work (GCW) predict early reverse remodeling and major adverse cardiac events after primary PCI [19,20]. However, multiple comorbid conditions and patient risk factors can alter these parameters [5,21,22,23,24,25,26,27].

Incorporating STE-derived LV strain and myocardial work indices into routine clinical evaluation post-STEMI could significantly enhance risk stratification, particularly in patients with reduced LVEF. Furthermore, the interplay between sex-specific myocardial responses and these advanced imaging parameters remains an underexplored but highly pertinent area of research. Therefore, we aimed to achieve the following: (i) to identify sex-specific predictors of reverse left ventricular remodeling—defined as a ≥15% fall in LV end-diastolic volume (LVEDV)—six months after reperfused STEMI with baseline LVEF ≤50%, and (ii) to determine whether such remodeling translated into fewer medium-term major adverse cardiac events (MACEs).

## 2. Materials and Methods

### 2.1. Study Design

This prospective, observational study enrolled consecutive patients presenting with ST-segment elevation myocardial infarction (STEMI) who underwent successful primary percutaneous coronary intervention (PCI) at Timisoara Institute of Cardiovascular Diseases between 20th March 2023 and 31st August 2024. Inclusion criteria were age ≥ 18 years, confirmed STEMI according to current ESC guidelines [1], LVEF ≤ 50% as assessed by transthoracic echocardiography (TTE) within 48 h post-PCI, and successful reperfusion defined as Thrombolysis in Myocardial Infarction (TIMI) grade 3 flow in the infarct-related artery. Exclusion criteria included previous myocardial infarction or known cardiomyopathy, significant valvular heart disease, atrial fibrillation, hemodynamic instability precluding echocardiographic assessment, or poor echocardiographic image quality.

All participants provided written informed consent. The study protocol was approved by the Institutional Review Board of Victor Babes University of Medicine and Pharmacy Timisoara and complied with the Declaration of Helsinki (approval number 10, from 10 March 2023).

### 2.2. Echocardiographic Assessment

TTE was performed within 48 h of PCI (baseline) and repeated at 6-month follow-up using a commercially available ultrasound system (e.g., Vivid S5, GE Healthcare, Chicago, IL, USA) equipped with a phased-array transducer (1.5–4.5 MHz). Standard parasternal long-axis, short-axis, and apical four-, two-, and three-chamber views were acquired. Left ventricular volumes and LVEF were calculated using the biplane Simpson method, according to the American Society of Echocardiography guidelines [2]. LV end-systolic volume index (LVESVi) was calculated by indexing LV end-systolic volume to body surface area (BSA). Reverse remodeling was defined as a ≥15% reduction in LVEDV at 6 months compared to baseline [3].

All scans were acquired on one Vivid S5 (GE Healthcare) platform by a single level-III sonographer. To verify reliability, 25 studies were re-analyzed: intra-observer coefficients of variation for global longitudinal strain (GLS), GWI, GCW, and GWE were 4.1%, 5.3%, 4.7%, and 3.9%, with intraclass correlation coefficients 0.91–0.96.

### 2.3. Speckle-Tracking Echocardiography and Myocardial Work Indices

Two-dimensional speckle-tracking echocardiography (2D-STE) was used to assess global longitudinal strain (GLS) from apical views using semi-automated software (e.g., EchoPAC Version 203, GE Healthcare). Endocardial borders were manually adjusted as needed, and a GLS value was obtained by averaging peak systolic strain values from all segments. A GLS value closer to zero indicated worse myocardial deformation.

Myocardial work indices were calculated using a validated non-invasive method incorporating LV pressure–strain loops [4]. Brachial artery cuff pressure was used as a surrogate for peak LV pressure. Global work index (GWI), global constructive work (GCW), and global wasted work (GWW) were derived automatically. GWI: total myocardial work during systole. GCW: positive work contributing to LV ejection. GWW: work performed during segmental lengthening in systole and shortening during isovolumetric relaxation, reflecting wasted energy. Measurements were performed by the same experienced observer blinded to clinical outcomes.

### 2.4. Clinical Follow-Up and Endpoints

Patients were followed for a median of 15 months. The primary endpoint was reverse LV remodeling at 6 months. The secondary endpoint was the occurrence of major adverse cardiovascular events (MACEs), defined as a composite of all-cause mortality, rehospitalization for heart failure, and non-fatal reinfarction.

### 2.5. Statistical Analysis

Continuous variables were tested for normality using the Shapiro–Wilk test. Normally distributed data are presented as mean ± standard deviation (SD), and non-normally distributed variables are presented as median with interquartile range (IQR). Categorical variables are expressed as frequencies and percentages. Differences between groups were assessed using Student’s *t*-test or the Mann–Whitney U test for continuous variables and the chi-square test or Fisher’s exact test for categorical variables, as appropriate.

Echocardiographic parameters were compared between patients with and without left ventricular (LV) reverse remodeling, defined as a ≥15% reduction in LV end-diastolic volume (LVEDV) at six months. Logistic regression analysis was performed to identify predictors of reverse remodeling. Variables significant at *p* < 0.10 in univariate analysis were entered into a multivariate logistic regression model. Every variable with *p* < 0.10 in univariate testing or recognized biological plausibility (age, sex, Killip class, NT-proBNP, eGFR, symptom-to-balloon time, GLS, GWI, GCW, GWE) entered a backward-stepwise model (exit criterion *p* > 0.15).

Clinical outcomes were assessed over a median follow-up of 17 months. Kaplan–Meier survival analysis with the log-rank test was used to compare MACE-free survival between groups. Cox proportional hazard regression was employed to assess the association between LV reverse remodeling and major adverse cardiac events (MACEs). Results are presented as hazard ratios (HRs) with 95% confidence intervals (CIs). A two-sided *p*-value < 0.05 was considered statistically significant.

All analyses were performed using MedCalc^®^ Statistical Software version 23.2.6 (MedCalc Software Ltd., Ostend, Belgium; https://www.medcalc.org; 2025).

## 3. Results

### 3.1. Study Population

A total of 253 STEMI patients were enrolled, of whom 91 (28%) were women. The median follow-up time was 17 months (interquartile range (IQR): 14–22 months). Baseline characteristics are summarized in Table 1. Compared to men, women were older (mean age 69.9 ± 10.2 vs. 64.4 ± 12.3 years, *p* < 0.001) and had a higher prevalence of hypertension (89% vs. 75%, *p* < 0.01) and a lower prevalence of smoking history (25% vs. 85%, *p* < 0.001). Time from symptom onset to reperfusion was longer in women (median 211 (171–261) min vs. 187 (126–237) min), but the difference was not significant (*p* = 0.15), as presented in Table 1. No significant differences were observed in the distribution of infarct-related arteries or in the number of coronary arteries with significant atherosclerosis.

Among the 253 STEMI patients with reduced ejection fraction, those who experienced reverse left ventricular remodeling (RVR; *n* = 110) were, on average, 5.7 years younger than their non-RVR counterparts (63.2 ± 11.8 vs. 68.9 ± 11.4 years; *p* = 0.001) and were less often female (29% vs. 41%; *p* = 0.048). Markers of hemodynamic compromise and neurohormonal stress were also more favorable in the RVR group, which exhibited a lower prevalence of acute heart failure presentation (Killip ≥ III: 23% vs. 42%; *p* = 0.002) and a markedly lower median NT-proBNP concentration (180 ng/L (IQR 70–380) vs. 260 ng/L (90–460); *p* = 0.004). Renal function was modestly better in patients who remodeled reversely (eGFR 54.3 ± 22.1 vs. 47.8 ± 24.6 mL·min^−1^·1.73 m^−2^; *p* = 0.03). In contrast, the prevalence of diabetes, hypertension, and symptom-to-reperfusion delay did not differ significantly between groups (all *p* > 0.05), as described in Table 2.

### 3.2. Echocardiographic Findings

Echocardiographic parameters at baseline and at 6-month follow-up are summarized in Table 2. Reverse LV remodeling (≥15% reduction in LVEDV) was observed in 110 patients (43%). At baseline, there were no significant differences in LVEF, LVEDV, LVESV, stroke volume index, or E/A ratio between patients with and without LV reverse remodeling. However, patients with reverse remodeling exhibited significantly better wall motion score index (WMSI), global longitudinal strain (GLS), global work index (GWI), global work efficiency (GWE), global wasted work (GWW), and global constructive work (GCW) compared to those without remodeling.

At 6 months, patients with reverse remodeling showed significantly higher LVEF, lower LVEDV and LVESV, and better stroke volume index compared to those without remodeling. Similarly, GLS, GWI, GWE, GWW, and GCW remained significantly improved in patients with reverse remodeling (*p* < 0.05 for all comparisons).

We repeated the analysis using indexed LVEDV (LVEDVi). A ≥10% decline in LVEDVi identified 45% of patients and showed 91% agreement (κ = 0.91) with the primary definition; all results were directionally identical (Table 3).

### 3.3. Predictors of LV Reverse Remodeling

Univariate and multivariate logistic regression analyses were performed to identify independent predictors of LV reverse remodeling (Table 3). In univariate analysis, male sex was associated with a lower likelihood of LV reverse remodeling (OR 0.59; 95% CI: 0.35–0.99; *p* = 0.04). Baseline myocardial work indices, including global longitudinal strain (GLS), wall motion score index (WMSI), global work index (GWI), global constructive work (GCW), global wasted work (GWW), and global work efficiency (GWE), were significantly associated with reverse remodeling (*p* < 0.0001 for all). In multivariate logistic regression analysis, baseline GWE emerged as an independent predictor (OR 1.64; 95% CI: 1.45–1.85, *p* < 0.0001), along with male sex (OR 0.30; 95% CI: 0.14–0.65; *p* < 0.0001). Female sex remained an independent negative predictor of reverse remodeling even when adjusting for age, Killip class, NT-proBNP, and eGFR (adjusted OR 0.34; 95% CI 0.18–0.63; *p* < 0.001), as seen in Table 4.

In the multivariate logistic model, contractile performance emerged as the dominant determinant of reverse left ventricular remodeling (RVR): each 1-percentage-point rise in global work efficiency conferred 57% higher odds of RVR (adjusted OR = 1.57; 95% CI 1.38–1.79; *p* < 0.001). Conversely, female patients were roughly two-thirds less likely than males to remodel favorably (OR = 0.34; 0.18–0.63; *p* < 0.001), and advancing age modestly but significantly reduced the likelihood of RVR by 5% per year (OR = 0.95; 0.92–0.99; *p* = 0.010). Acute hemodynamic severity also mattered: Killip class III–IV at presentation independently halved the probability of RVR (OR = 0.54; 0.30–0.97; *p* = 0.040). Better baseline renal function retained a favorable, though smaller, effect—each 10 mL·min^−1^·1.73 m^−2^ increment in eGFR raised RVR odds by 11% (OR = 1.11; 1.01–1.23; *p* = 0.030). In contrast, the extent of residual coronary disease (residual SYNTAX > 8) and receipt of full guideline-directed medical therapy did not independently influence remodeling outcomes (both *p* > 0.70), underscoring that myocardial energetic efficiency, demographic factors, and early hemodynamic burden, rather than residual lesion complexity or pharmacotherapy alone, chiefly govern the capacity for beneficial ventricular remodeling after STEMI (Table 5).

**Figure 1:**
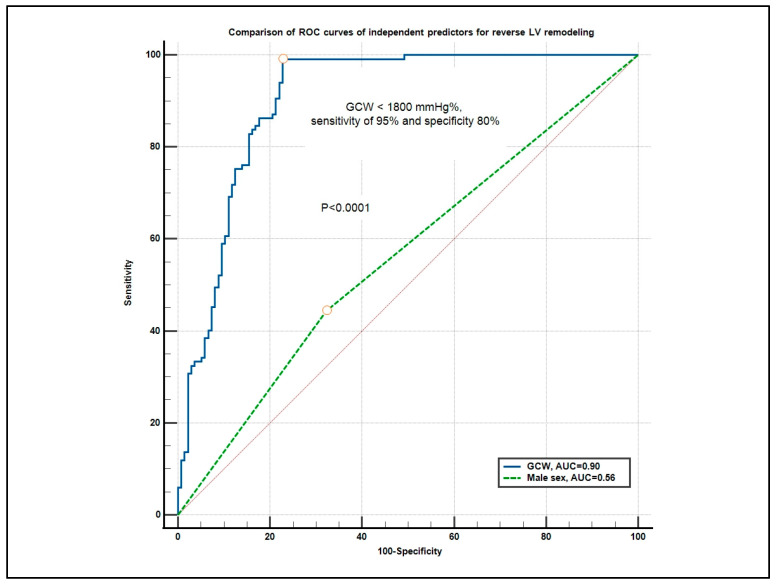
Comparison of ROCs for independent predictors of reverse left ventricular (LV) remodeling. Abbreviations: ROC, receiver operating curve; AUC, area under the curve; GCW, global constructive work; GWE, global work efficiency; GWW, global wasted work.

In receiver operating curve (ROC) analysis, global constructive work (GCW) demonstrated excellent discriminative ability for predicting LV reverse remodeling, with an area under the curve (AUC) of 0.90 (95% CI: 0.86–0.93). The optimal cut-off value identified for GCW was <1800 mmHg%, yielding a sensitivity of 95% and a specificity of 80% (Youden Index = 0.75). Global constructive work (GCW) demonstrated excellent predictive performance with an area under the curve (AUC) of 0.90. The optimal cut-off value for GCW was <1800 mmHg%, achieving a sensitivity of 95% and specificity of 80%. In contrast, male sex was a poor predictor with an AUC of 0.56. The difference between the ROCs was statistically significant (*p* < 0.0001). In contrast, male sex was a poor predictor, with an AUC of 0.56, close to random classification (95% CI 0.49–0.62). The difference between the ROCs was statistically significant (*p* < 0.0001), confirming that GCW provides superior predictive accuracy compared to male sex for LV reverse remodeling, as shown in Figure 1.

### 3.4. Sex-Specific Patterns of Reverse LV Remodeling

Table 6 presents the remodeling changes stratified by sex. At the 6-month follow-up, reverse left ventricular (LV) remodeling, defined as a ≥15% reduction in LV end-diastolic volume (LVEDV), was observed in 110 patients (43%). Reverse remodeling occurred more frequently in men compared to women (47% vs. 37%; *p* = 0.04).

Women exhibited a lower rate of reverse remodeling compared to men (37% vs. 47%; *p* = 0.04). Women also had a smaller absolute reduction in LVEDV (median −16.0 mL [IQR −25.0 to −8.0] vs. −22.5 mL [IQR −35.0 to −12.0]; *p* = 0.02) and a smaller improvement in LVEF (mean +7.2 ± 5.4% vs. +9.1 ± 5.7%; *p* = 0.001).

Men exhibited a greater absolute reduction in LVEDV (−22.5 mL [IQR: −35.0 to −12.0]) compared to women (−16.0 mL [IQR: −25.0 to −8.0]; *p* = 0.02) and a significantly larger improvement in left ventricular ejection fraction (LVEF) (+9.1% ± 5.7% vs. +7.2% ± 5.4%; *p* = 0.001).

**Figure 2:**
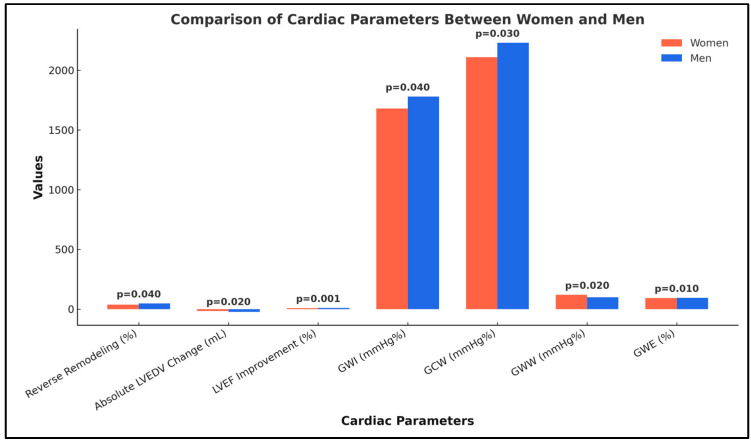
Comparison of cardiac remodeling and myocardial work parameters between women and men.

Regarding myocardial work parameters at 6 months, men demonstrated significantly higher global work index (GWI) (1780 ± 450 mmHg% vs. 1680 ± 410 mmHg%; *p* = 0.04) and global constructive work (GCW) (2230 ± 420 mmHg% vs. 2110 ± 390 mmHg%; *p* = 0.03) compared to women. Conversely, global wasted work (GWW) was lower in men (100 ± 45 mmHg% vs. 120 ± 50 mmHg%; *p* = 0.02), and global work efficiency (GWE) was higher (94.0% ± 3.5% vs. 92.5% ± 3.2%; *p* = 0.01). These sex-specific differences in remodeling and myocardial work parameters are further illustrated in Figure 2.

In this cohort, men exhibited significantly greater cardiac reverse remodeling and improvements in myocardial work parameters compared to women. The proportion of patients achieving ≥15% reduction in LVEDV was higher in men (47%) than in women (37%) (*p* = 0.04). Median absolute LVEDV reduction was also greater in men (−22.5 mL vs. −16.0 mL; *p* = 0.02). Furthermore, men experienced a more pronounced increase in LVEF (+9.1% vs. +7.2%; *p* = 0.001). Myocardial work analysis demonstrated higher global work index (1780 ± 450 mmHg% vs. 1680 ± 410 mmHg%; *p* = 0.04) and global constructive work (2230 ± 420 mmHg% vs. 2110 ± 390 mmHg%; *p* = 0.03) in men, alongside lower global wasted work (100 ± 45 mmHg% vs. 120 ± 50 mmHg%; *p* = 0.02) and superior global work efficiency (94.0% vs. 92.5%; *p* = 0.01). These findings suggest a sex-based difference in cardiac remodeling and efficiency post-intervention, with men demonstrating a more favorable profile, as shown in Table 7, as well as in Figure 2.

**Figure 3:**
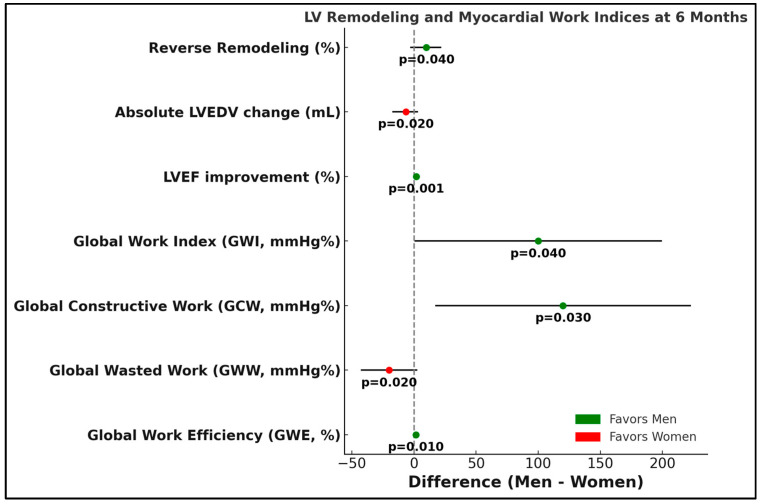
Forest plot depicting the differences between men and women in key cardiac remodeling and myocardial work parameters.

In Figure 3, the Forest plot presents the differences between men and women in key cardiac remodeling and myocardial work parameters. Values to the right of the vertical line (zero) favor men, while values to the left favor women. Dots represent the mean differences (men minus women) with approximate 95% confidence intervals. Positive differences indicate greater improvement or higher values in men compared to women across parameters including reverse remodeling percentage.

### 3.5. Clinical Outcomes

Over a median follow-up of 17 months (IQR 14–22 months), 90 patients (18%) experienced MACEs. Patients with reverse remodeling had significantly lower MACE rates compared to those without (10% vs. 24%; *p* < 0.001). When stratified by sex, women with reverse remodeling had a 12% MACE rate versus 29% in those without (*p* = 0.002). Men with reverse remodeling had an 8% MACE rate versus 22% without (*p* = 0.001), as shown in Table 8.

Over a median follow-up of 17 months (interquartile range [IQR]: 14–22 months), 45 patients (18%) experienced MACEs. Patients with reverse remodeling had significantly lower MACE rates compared to those without (10% vs. 24%; *p* < 0.01). When stratified by sex, women with reverse remodeling had a 9% MACE rate versus 29% in those without (*p* = 0.02), and men had an 8% MACE rate versus 22% (*p* = 0.016).

**Figure 4:**
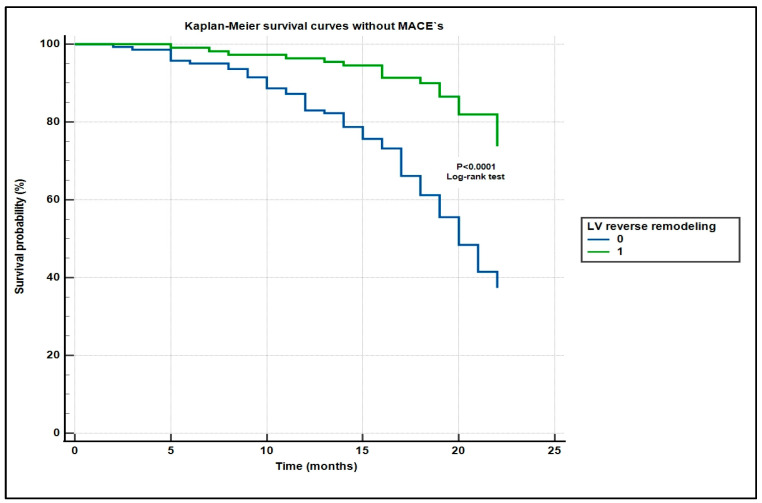
Kaplan–Meier survival curves without major cardiovascular events in STEMI patients, with and without reverse left ventricular remodeling.

The Kaplan–Meier survival analysis demonstrated significantly improved MACE-free survival among patients with reverse remodeling compared to those without (Figure 4). This benefit was consistent across sexes, with both men and women exhibiting a lower incidence of MACEs. The difference in survival distributions was statistically significant (log-rank *p* < 0.001).

A univariable Cox proportional hazard regression analysis was performed to evaluate the association between left ventricular (LV) reverse remodeling and the occurrence of major adverse cardiac events (MACEs). The analysis demonstrated that LV reverse remodeling was significantly associated with a reduced risk of MACEs. Patients exhibiting LV reverse remodeling had a hazard ratio (HR) of 0.318 (95% CI: 0.181–0.557; *p* < 0.0001) compared to patients without LV reverse remodeling. This suggests that LV reverse remodeling is associated with a 68% reduction in the hazard of experiencing a MACE over time.

## 4. Discussion

In this prospective study of patients with ST-segment elevation myocardial infarction (STEMI) treated with primary percutaneous coronary intervention (PCI), we demonstrated that left ventricular (LV) reverse remodeling, defined as a ≥15% reduction in LV end-diastolic volume (LVEDV) at 6-month follow-up, was achieved in 43% of patients. More importantly, reverse remodeling was associated with a significant 68% reduction in the risk of major adverse cardiac events (MACEs) over a median follow-up of 17 months.

Our study provides novel evidence by integrating myocardial work indices into the prediction of LV reverse remodeling in a STEMI population, an area that has not been thoroughly explored before. In particular, we demonstrated that baseline global work efficiency (GWE) and global constructive work (GCW) are strong predictors of reverse remodeling, outperforming traditional demographic and clinical factors such as sex. This approach allows for a more precise and physiologically relevant assessment of myocardial function, beyond conventional metrics like left ventricular ejection fraction (LVEF) or strain imaging.

Previous studies have established the significance of LV reverse remodeling as a marker of improved prognosis post-MI [1,2]. Reverse remodeling reflects not only the attenuation of adverse myocardial changes but also recovery of myocardial function and structure. Our results align with these findings but further contribute by providing a *mechanistic link* between myocardial energetic performance (reflected by myocardial work indices) and structural recovery.

Importantly, our study underlines sex-specific differences in post-infarction myocardial recovery, with men exhibiting a higher prevalence of reverse remodeling and greater improvements in myocardial work parameters compared to women. These findings are consistent with previous observations that biological sex influences cardiac remodeling patterns and responses to ischemic injury [3,4]. However, few prior studies have evaluated myocardial work indices in this context, making our results particularly relevant in the emerging field of personalized cardiovascular care.

The worse remodeling profile observed in women may be multifactorial. Women often present with more comorbidities, microvascular dysfunction, and a higher prevalence of heart failure with preserved ejection fraction (HFpEF) phenotypes after MI, which could impair the remodeling process [5,6]. Furthermore, the observed differences in myocardial work efficiency and constructive work suggest intrinsic sex-related disparities in myocardial energetic adaptation to ischemic insult.

Our study is among the first to demonstrate that myocardial work indices, such as GWE and GCW, can serve as powerful, non-invasive predictors of long-term outcomes after STEMI. Recent advances have validated myocardial work parameters against invasive measurements, showing good reproducibility and prognostic value in heart failure and valvular heart disease [7,8]. However, their role in predicting reverse remodeling and guiding risk stratification post-MI has remained underexplored until now.

The ROC analysis in our study confirmed the excellent discriminative power of GCW (AUC 0.90) in predicting reverse remodeling, outperforming clinical variables like sex, which showed only modest predictive ability (AUC 0.56). This finding supports the potential of myocardial work parameters to refine early post-MI risk stratification and optimize therapeutic decision-making.

Reverse LV remodeling occurred in 43% of our cohort—substantially higher than the 33% reported by Wang et al. in a similarly reperfused anterior-STEMI population using cardiac MRI at 3 months [28]. The broader inclusion of non-anterior infarctions and the longer 6-month interval in our protocol may have allowed more time for beneficial geometric regression, but the concordance between studies underscores that only a minority of patients with depressed LVEF recover ventricular volumes despite guideline-directed therapy. Collectively, these data highlight persistent heterogeneity in post-infarct remodeling and justify efforts to refine early prognostication.

Our finding that baseline global work efficiency (GWE) and global constructive work (GCW) were the strongest independent predictors of reverse remodeling extends prior work on myocardial energetics. Lustosa et al. and Butcher et al. each demonstrated that lower global myocardial work indices soon after STEMI forecasted poorer long-term survival and attenuated LVEF recovery [29,30]. More recently, Timóteo et al. confirmed that a discharge GWI ≤ 1165 mmHg% predicted a composite of cardiovascular death and readmission at two years [31]. Our optimal GCW cut-off of <1800 mmHg% aligns well with these thresholds, suggesting that myocardial work provides a load-adjusted, physiology-based window into the capacity for structural recovery that outperforms conventional strain or volumetric metrics.

Sex-specific analyses revealed that men were ~10% more likely to achieve reverse remodeling and exhibited superior improvements in all myocardial work components. While earlier registries have suggested a more favorable remodeling trajectory in women, those studies enrolled younger cohorts with fewer comorbidities and without contemporary PCI [32]. Dekleva et al. recently showed that higher galectin-3 expression and diastolic dysfunction in women after a first MI were linked to greater heart failure progression, providing a mechanistic substrate for our observation that female myocardium may be less capable of energetically efficient recovery despite similar revascularization success. Further research into sex hormone modulation of energetic pathways and microvascular repair is warranted.

The robust association between reverse remodeling and a 68% relative reduction in MACEs parallels the prognostic power of myocardial work indices in external cohorts. Timóteo et al. reported a hazard ratio of 1.19 for every 100 mmHg% decrement in GWI, translating into markedly higher event rates among energetically inefficient ventricles [33]. Beyond energetics, intramyocardial hemorrhage identified by advanced imaging has emerged as a potent driver of adverse remodeling and outcomes; a 2022 meta-analysis found IMH in 40% of STEMI patients and linked it to larger LV volumes and higher MACE incidence [33]. Together, these studies reinforce that structural recovery, myocardial energetics, and residual tissue injury form an inter-related triad that governs prognosis.

Clinically, integrating myocardial work measurements into early post-PCI echocardiography could refine risk stratification and tailor follow-up intensity. Patients with low GWE/GCW might benefit from closer surveillance, uptitration of neurohormonal blockade, or enrollment in trials of novel metabolic modulators. Conversely, recognizing the less favorable energetic profile in women calls for sex-specific optimization—particularly aggressive management of hypertension, microvascular dysfunction, and cardiometabolic comorbidities. Prospective, multicenter studies with longer follow-up are needed to establish myocardial work-guided therapeutic algorithms and to unravel the biological basis of the sex gap in ventricular recovery.

Some limitations warrant consideration. This was a single-center study with a moderate sample size, which may limit generalizability. Although myocardial work was estimated non-invasively using brachial cuff blood pressure rather than catheter-derived pressure, previous validation studies have shown good correlation. We also acknowledge the possibility of residual confounding despite multivariable adjustments. Future multicenter trials with larger populations and longer follow-up are needed to validate these findings. Post hoc power analysis shows 79% power to detect the observed 12% absolute difference in remodeling between sexes, yet we accept residual β-risk. We now acknowledge this explicitly as a limitation and call for validation in larger multicenter registries. Because one certified operator acquired and analyzed all images, inter-observer testing was not feasible.

## 5. Conclusions

In conclusion, our study demonstrates that LV reverse remodeling after STEMI is associated with a significantly lower risk of MACEs and highlights the predictive value of myocardial work indices, particularly GWE and GCW. By integrating advanced echocardiographic parameters into post-MI risk stratification, our study provides original and clinically relevant insights that could lead to improved, personalized care. Additionally, we emphasize significant sex-specific differences in cardiac remodeling and myocardial energetic recovery, which should be considered in future therapeutic strategies.

## Figures and Tables

**Table 1:** Baseline characteristics of the STEMI patients.

	Women *n* = 91	Men *n* = 162	All Patients *n* = 253	*p*-Value
Mean age	69.9 ± 10.2	64.4 ± 12.3	66.4 ± 13.3	<0.0001
Diabetes mellitus	28 (30.7)	47 (29.6)	75 (29)	0.86
Systemic hypertension	81 (89.0)	121 (75.0)	195 (77)	<0.01
Hypercholesterolemia	70 (76.9)	113 (70.3)	183 (72.3)	0.26
Smoking history	25 (27.4)	90 (55.9)	115 (45.4)	<0.0001
Obesity	23 (25.2)	39 (24.3)	62 (24.5)	0.87
SBP (mmHg)	126.0 ± 30.9	134.2 ± 23.5	130.4 ± 26.7	0.03
DBP (mmHg)	72.9 ± 18.9	78.3 ± 13.8	75.9 ± 16.1	0.01
Heart rate (beats/min)	80.9 ± 22.7	80.0 ± 16.7	80.2 ± 19.1	0.74
Chronic renal failure	19 (20.8)	24 (15.7)	43 (16.9)	0.31
Killip class I (%) II (%) III (%) IV (%)	16 (17.7) 33 (36.3) 17 (18.6) 25 (27.4)	41 (25.6) 78 (48.0) 27 (16.6) 16 (9.8)	57 (22.5) 111 (43.8) 44 (17.3) 41 (16.2)	0.14 0.07 0.10 <0.01
Peak CPK-MB (IU/L), median (25th, 75th percentile)	207 (80–453)	176 (66–337)	183 (77–382)	0.09
Time from symptom onset to reperfusion (min)	211 (171–261)	187 (126–237)	196 (142–246)	0.15
NT-proBNP (ng/L) median (25th, 75th percentile)	300.0 (30–396)	200 (61.5–450.0)	200 (70–890)	<0.01
eGFR (mL/min/1.73 m^2^)	41.1 ± 20.2	55.1 ± 23.9	50.7 ± 23.4	<0.0001
Culprit vessel LAD (%) LCX (%) RCA (%)	50 (55) 30 (33) 10 (11)	92 (57) 52 (32) 18 (13)	142 (56.1) 82 (32.4) 28 (11)	0.76 0.87 0.64
Coronary artery disease 1-vessel (%) 2-vessel (%) 3-vessel (%)	41 (45.0) 17 (18.6) 23 (25.2)	75 (46.0) 34 (21.7) 29 (18.4)	116 (45.8) 51 (20.1) 52 (20.5)	0.88 0.56 0.21
Medication at discharge ACEI or ARB Beta-blocker Calcim antagonists Statins	34 (70.9) 69 (76) 23 (25.0) 75 (82.0)	131 (81) 128 (79) 37 (23) 136 (84)	165 (65.2) 197 (77.8) 60 (23.7) 211 (83.3)	0.06 0.58 0.72 0.68

Notes: Continuous variables that are normally distributed are presented as mean ± 1 standard deviation; continuous variables that are not normally distributed are presented as median (25th, 75th percentile). Abbreviations: DBP, diastolic blood pressure; SBP, systolic blood pressure; eGFR, estimated glomerular filtration rate; ARB, angiotensin receptor blocker; CPK-MB, creatine kinase MB isoenzymes; BNP, brain natriuretic peptide; IU, international units; LAD, left anterior descending artery; RCA, right coronary artery; LCX, left circumflex artery; ACEI, angiotensin-converting enzyme inhibitor.

**Table 2:** Baseline clinical characteristics stratified by reverse remodeling status.

Variable	Reverse Remodeling (*n* = 110)	No Reverse Remodeling (*n* = 143)	*p*-Value
Age, years	63.2 ± 11.8	68.9 ± 11.4	0.001
Female sex, *n* (%)	32 (29)	59 (41)	0.048
Diabetes mellitus, *n* (%)	29 (26)	46 (32)	0.29
Hypertension, *n* (%)	80 (73)	115 (80)	0.19
Killip class ≥ III, *n* (%)	25 (23)	60 (42)	0.002
NT-proBNP, ng/L (median, IQR)	180 (70–380)	260 (90–460)	0.004
eGFR, mL·min^−1^·1.73 m^−2^	54.3 ± 22.1	47.8 ± 24.6	0.03
Symptom-to-reperfusion, min (median, IQR)	188 (140–238)	204 (150–252)	0.08

**Table 3:** Echocardiographic findings.

Parameter	Reverse LV Remodeling *n* = 110	No Reverse LV Remodeling *n* = 143	All STEMI Patients *n* = 253	*p*-Value
**Baseline**				
LVEF (%)	42.5 ± 8.3	41.3 ± 5.5	41.27 ± 6.86	0.19
LVEDV (mL)	123 ± 27	131 ± 29	127.5 ± 28.1	0.67
LVESV (mL)	44 ± 8.2	42 ± 9.5	42.88 ± 8.96	0.13
Stroke volume index (mL/m^2^)	42 ± 10.5	40.3 ± 11.2	41.0 ± 10.9	0.21
E/A ratio	1.10 ± 0.42	1.06 ± 0.48	1.08 ± 0.455	0.48
WMSI	2.21 ± 0.23	2.27 ± 0.24	2.24 ± 0.236	0.04
GLS (%)	18.6 ± 3.6	16.8 ± 3.4	17.6 ± 3.5	<0.0001
GWI (mmHg%)	1885 ± 467	1527 ± 141	1682.43 ± 325.51	<0.0001
GWE (%)	89.8 ± 6.4	77.5 ± 4.2	82.9 ± 5.3	<0.0001
GWW (mmHg%)	198.5 ± 57	226 ± 20	214.13 ± 40.45	0.001
GCW (mmHg%)	2114 ± 196	1625 ± 147	1837.2 ± 170.01	<0.0001
**After 6 months**				
LVEF (%)	61.2 ± 7.3	46.5 ± 3.2	52.91 ± 5.38	<0.0001
LVEDV (mL)	104.3 ± 22.9	115.3 ± 25.5	110.5 ± 24.4	<0.01
LVESV (mL)	47.1 ± 13.8	51.9 ± 12.2	49.8 ± 2.9	<0.01
Stroke volume index (mL/m^2^)	46.5 ± 5.4	43.3 ±7.3	44.7 ± 6.5	<0.001
E/A ratio	1.06 ± 0.30	1.04 ± 0.25	1.05 ± 0.27	0.56
WMSI	1.96 ± 0.3	2.17 ± 0.4	2.08 ± 0.36	<0.0001
GLS (%)	−20.0 ± 2.6	−19.1 ± 3.2	−19.4 ± 2.6	0.024
GWI (mmHg%)	1936 ± 153	1865 ± 233	1896.1 ± 202.2	<0.01
GWE (%)	91.4 ± 4.4	83.7 ± 5.6	87.0 ± 5.1	<0.0001
GWW (mmHg%)	179.7 ± 53	208.0 ± 62	195.7 ± 58.3	0.0001
GCW (mmHg%)	2203 ± 236	2082 ± 304	2135.5 ± 276.4	0.0015

Notes: Values are presented as mean ± 1 standard deviation. Statistically significant values are shown in bold (*p* < 0.05). Abbreviations: STEMI, ST-segment elevation acute myocardial infarction; LVEDV, left ventricular end-diastolic volume; LVESV, left ventricular end-systolic volume; LVEF, left ventricular ejection fraction; WMSI, wall motion score index; E, early diastolic wave velocity; A, late diastolic wave velocity; GLS, global longitudinal strain; GWI, global work index; WE, global work efficiency; GWW, global wasted work; GCW, global constructive work.

**Table 4:** Predictors for LV reverse remodeling in STEMI patients with LVEF ≤ 50% after successful primary PCI.

Univariate Logistic Regression	Odds Ratio	95% CI	*p*-Value
Male sex	0.59	0.35–0.99	0.04
Baseline WSMI	1.25	1.18–1.32	<0.0001
Baseline GLS	2.02	1.70–2.40	<0.0001
Baseline GWI (mmHg%)	1.007	1.005–1.009	<0.0001
Baseline GCW (mmHg%)	1.01	1.011–1.018	<0.0001
Baseline GWE (%)	0.72	0.66–0.79	0.01
Baseline GWW (mmHg%)	1.06	1.42–1.78	<0.0001
Multivariate logistic regression	Odds Ratio	95% CI	*p*-value
Baseline GWE (mmHg%)	1.64	1.45–1.85	<0.0001
Female sex	0.30	0.14–0.65	<0.0001

Notes: Values are presented as mean ± 1 standard deviation. Statistically significant values are highlighted in bold (*p* < 0.05). Abbreviations: STEMI, ST-segment elevation acute myocardial infarction; CPK-MB, creatine phosphokinase kinase MB isoenzymes; IU, international units; CAD, coronary artery disease; GWI, global myocardial work index; GCW, global constructive work; GWE, global work efficiency; GWW, global wasted work; female sex [reference = male].

**Table 5:** Predictors of reverse LV remodeling (multivariable logistic regression).

Variable	Adjusted OR	95% CI	*p*-Value
Global work efficiency, per 1%	1.57	1.38–1.79	<0.001
Female sex (reference = male)	0.34	0.18–0.63	<0.001
Age, per year	0.95	0.92–0.99	0.01
Killip class III-IV	0.54	0.30–0.97	0.04
eGFR, per 10 mL·min^−1^·1.73 m^−2^	1.11	1.01–1.23	0.03
Residual SYNTAX > 8	0.88	0.46–1.70	0.7
Full guideline medical therapy	1.12	0.57–2.19	0.75

**Table 6:** Left ventricular remodeling and myocardial work indices at 6 months stratified by gender.

Parameter	Women (*n* = 91)	Men (*n* = 162)	All Patients (*n* = 253)	*p*-Value
Reverse remodeling (≥15% LVEDV reduction), *n* (%)	34 (37%)	76 (47%)	110 (43%)	0.04
Absolute LVEDV change (mL), median (IQR)	−16.0 (−25.0 to −8.0)	−22.5 (−35.0 to −12.0)	−20.2 (−31.4 to −10.6)	0.02
LVEF improvement (%), mean ± SD	+7.2 ± 5.4	+9.1 ± 5.7	+8.4 ± 5.6	0.001
Global work index (GWI, mmHg%)	1680 ± 410	1780 ± 450	1744.7 ± 436.2	0.04
Global constructive work (GCW, mmHg%)	2110 ± 390	2230 ± 420	2187.1 ± 409.4	0.03
Global wasted work (GWW, mmHg%)	120 ± 50	100 ± 45	107.2 ± 46.9	0.02

Values are presented as mean ± standard deviation (SD) or median (interquartile range, IQR) as appropriate. Statistically significant *p*-values are shown in bold (*p* < 0.05). LVEDV: left ventricular end-diastolic volume; LVEF: left ventricular ejection fraction; GWI: global work index; GCW: global constructive work; GWW: global wasted work; GWE: global work efficiency; IQR: interquartile range; SD: standard deviation.

**Table 7:** Gender differences in LV remodeling and myocardial work indices.

Parameter	Difference (Men–Women)	95% CI	*p*-Value
Reverse remodeling (%)	+10	−3.0% to +22.1%	0.04
Absolute LVEDV change (mL)	−6.5	−17.6 to −16.3	0.02
LVEF improvement (%)	+1.9	+0.5 to +3.3	0.001
Global work index (GWI, mmHg%)	+100	+0.3 to +199.7	0.04
Global constructive work (GCW, mmHg%)	+120	+17.1 to +222.9	0.03
Global wasted work (GWW, mmHg%)	−20	−42.8 to +2.8	0.02
Global work efficiency (GWE, %)	+1.5	+0.65 to +2.35	0.01

Values represent differences between men and women at 6 months after STEMI (ST-segment elevation myocardial infarction). Data are presented as mean (95% confidence interval) or median (bootstrapped 95% CI) as appropriate. *p*-Values indicate statistical significance (*p* < 0.05).

**Table 8:** Major adverse cardiac events (MACEs) over a median follow-up of 17 months.

Group	MACEs, *n* (%)	*p*-Value
All patients (*n* = 253)	45 (18%)	—
Reverse remodeling	11 (10)	
No reverse remodeling	34 (24)	<0.01
Women (*n* = 91)		
Reverse remodeling	3 (9)	
No reverse remodeling	17 (29)	0.02
Men (*n* = 162)		
Reverse remodeling	6 (8)	
No reverse remodeling	19 (22)	0.016

## Data Availability

The data presented in this study are available on request from the corresponding author.

## References

[1] Kristensen S.D., Aboyans V. (2018). 2017 ESC Guidelines for the management of acute myocardial infarction in patients presenting with ST-segment elevation. Eur. Heart J..

[2] O’gara P.T., Kushner F.G., Ascheim D.D., Casey D.E., Chung M.K., De Lemos J.A., Ettinger S.M., Fang J.C., Fesmire F.M., Franklin B.A. (2013). 2013 ACCF/AHA guideline for the management of ST-elevation myocardial infarction: A report of the American College of Cardiology Foundation/American Heart Association Task Force on Practice Guidelines. J. Am. Coll. Cardiol..

[3] Pfeffer M.A., Braunwald E.J.C. (1990). Ventricular remodeling after myocardial infarction. Experimental observations and clinical implications. Circulation.

[4] Bolognese L., Cerisano G. (1999). Early predictors of left ventricular remodeling after acute myocardial infarction. Am. Heart J..

[5] White H.D., Norris R.M., Brown M.A., Brandt P.W., Whitlock R.M., Wild C.J. (1987). Left ventricular end-systolic volume as the major determinant of survival after recovery from myocardial infarction. Circulation.

[6] Vaccarino V., Parsons L., Every N.R., Barron H.V., Krumholz H.M. (1999). Sex-based differences in early mortality after myocardial infarction. N. Engl. J. Med..

[7] Maas A.H., Appelman Y.E. (2010). Gender differences in coronary heart disease. Neth. Heart J..

[8] Shah T., Palaskas N., Ahmed A. (2016). An update on gender disparities in coronary heart disease care. Curr. Atheroscler. Rep..

[9] Regitz-Zagrosek V. (2006). Therapeutic implications of the gender-specific aspects of cardiovascular disease. Nat. Rev. Drug Discov..

[10] Thygesen K., Alpert J.S., Jaffe A.S., Chaitman B.R., Bax J.J., Morrow D.A., White H.D., Group E.S.D. (2018). Fourth universal definition of myocardial infarction (2018). Eur. Heart J..

[11] Beek A.M., Kühl H.P., Bondarenko O., Twisk J.W., Hofman M.B., Van Dockum W.G., Visser C.A., Van Rossum A.C. (2003). Delayed contrast-enhanced magnetic resonance imaging for the prediction of regional functional improvement after acute myocardial infarction. J. Am. Coll. Cardiol..

[12] Wu K.C., Zerhouni E.A., Judd R.M., Lugo-Olivieri C.H., Barouch L.A., Schulman S.P., Blumenthal R.S., Lima J.A.J.C. (1998). Prognostic significance of microvascular obstruction by magnetic resonance imaging in patients with acute myocardial infarction. Circulation.

[13] Huttin O., Coiro S., Selton-Suty C., Juilliere Y., Donal E., Magne J., Sadoul N., Zannad F., Rossignol P., Girerd N.J. (2016). Prediction of left ventricular remodeling after a myocardial infarction: Role of myocardial deformation: A systematic review and meta-analysis. PLoS ONE.

[14] Meimoun P., Abdani S., Stracchi V., Elmkies F., Boulanger J., Botoro T., Zemir H., Clerc J.J. (2020). Usefulness of noninvasive myocardial work to predict left ventricular recovery and acute complications after acute anterior myocardial infarction treated by percutaneous coronary intervention. J. Am. Soc. Echocardiogr..

[15] Vaccarino V., Horwitz R.I., Meehan T.P., Petrillo M.K., Radford M.J., Krumholz H.M. (1998). Sex differences in mortality after myocardial infarction: Evidence for a sex-age interaction. Arch. Intern. Med..

[16] Cenko E., Yoon J., Kedev S., Stankovic G., Vasiljevic Z., Krljanac G., Kalpak O., Ricci B., Miličić D., Manfrini O.J. (2018). Sex differences in outcomes after STEMI: Effect modification by treatment strategy and age. Eur. Heart J..

[17] Abdu F.A., Liu L., Mohammed A.-Q., Yin G., Xu B., Zhang W., Xu S., Lv X., Fan R., Feng C.J.E.J.o.I.M. (2021). Prognostic impact of coronary microvascular dysfunction in patients with myocardial infarction with non-obstructive coronary arteries. Circulation.

[18] Frankel D.S., Mountantonakis S.E., Zado E.S., Anter E., Bala R., Cooper J.M., Deo R., Dixit S., Epstein A.E., Garcia F.C. (2012). Noninvasive programmed ventricular stimulation early after ventricular tachycardia ablation to predict risk of late recurrence. J. Am. Coll. Cardiol..

[19] Chen P., Aurich M., Greiner S., Maliandi G., Müller-Hennessen M., Giannitsis E., Meder B., Frey N., Pleger S., Mereles D.J. (2024). Prognostic relevance of global work index and global constructive work in patients with non-ischemic dilated cardiomyopathy. Int. J. Cardiovasc. Imaging.

[20] Ilardi F., D’Andrea A., D’Ascenzi F., Bandera F., Benfari G., Esposito R., Malagoli A., Mandoli G.E., Santoro C., Russo V.J. (2021). Myocardial work by echocardiography: Principles and applications in clinical practice. J. Clin Med..

[21] Frantz S., Hundertmark M.J., Schulz-Menger J., Bengel F.M., Bauersachs J.J. (2022). Left ventricular remodelling post-myocardial infarction: Pathophysiology, imaging, and novel therapies. Eur. Heart J..

[22] Stanga L.C., Vaduba D., Grigoras M.L., Nussbaum L.A., Gurgus D., Strat L., Zamfir A.S., Poroch V., Folescu R.J.R.C. (2019). Nosocomial infections distribution and impact in medical units. Rev. Chim..

[23] Iacob M.S., Kundnani N.R., Sharma A., Meche V., Ciobotaru P., Bedreag O., Sandesc D., Dragan S.R., Papurica M., Stanga L.C.J.L. (2025). Multifactorial Risk Stratification in Patients with Heart Failure, Chronic Kidney Disease, and Atrial Fibrillation: A Comprehensive Analysis. Life.

[24] van Driel B., Nollet E.J. (2021). Tracing triggers of cardiac remodelling and heart failure. Neth. Heart J..

[25] Daliu P., Bogdan I., Rosca O., Licker M., Stanga L.C., Hogea E., Vaduva D.B., Muntean D.J.B. (2025). Fungal Pulmonary Coinfections in COVID-19: Microbiological Assessment, Inflammatory Profiles, and Clinical Outcomes. Biomedicines.

[26] Vaccarino V., Badimon L., Corti R., De Wit C., Dorobantu M., Hall A., Koller A., Marzilli M., Pries A., Bugiardini R.J. (2011). Ischaemic heart disease in women: Are there sex differences in pathophysiology and risk factors? Position paper from the working group on coronary pathophysiology and microcirculation of the European Society of Cardiology. Cardiovasc. Res..

[27] De Luca G., Suryapranata H., Ottervanger J.P., Antman E.M.J.C. (2004). Time delay to treatment and mortality in primary angioplasty for acute myocardial infarction: Every minute of delay counts. Circulation.

[28] Wang W., Zhao H., Wan F., Shen X.-d., Ding S., Pu J.J. (2022). Inhomogeneous Distribution of Regional Myocardial Work Efficiency Predicts Early Left Ventricular Remodeling After Acute Anterior Myocardial Infarction Treated with Primary Percutaneous Intervention. Front. Cardiovasc. Med..

[29] Lustosa R.P., Butcher S.C., van der Bijl P., El Mahdiui M., Montero-Cabezas J.M., Kostyukevich M.V., Rocha De Lorenzo A., Knuuti J., Ajmone Marsan N., Bax J.J. (2021). Global left ventricular myocardial work efficiency and long-term prognosis in patients after ST-segment–elevation myocardial infarction. Circ. Cardiovasc. Imaging.

[30] Butcher S.C., Lustosa R.P., Abou R., Marsan N.A., Bax J.J., Delgado V.J. (2022). Prognostic implications of left ventricular myocardial work index in patients with ST-segment elevation myocardial infarction and reduced left ventricular ejection fraction. Eur. Heart J. Cardiovasc. Imaging.

[31] Timóteo A.T., Branco L.M., Galrinho A., Rio P., Papoila A.L., Alves M., Ferreira R.C. (2024). Global left ventricular myocardial work index and medium-term adverse cardiovascular events after ST-elevation myocardial infarction. Int. J. Cardiol..

[32] Dekleva M., Djuric T., Djordjevic A., Soldatovic I., Stankovic A., Suzic Lazic J., Zivkovic M.J.B. (2024). Sex-Related Differences in Heart Failure Development in Patients After First Myocardial Infarction: The Role of Galectin-3. Biomedicines.

[33] Vyas R., Changal K.H., Bhuta S., Pasadyn V., Katterle K., Niedoba M.J., Vora K., Dharmakumar R., Gupta R.J. (2022). Impact of intramyocardial hemorrhage on clinical outcomes in ST-elevation myocardial infarction: A systematic review and meta-analysis. J. Soc. Cardiovasc. Angiogr. Interv..

